# A Comparison of the ASEBA Adult Self Report (ASR) and the Brief Problem Monitor (BPM/18-59)

**DOI:** 10.1007/s10519-020-10001-3

**Published:** 2020-05-17

**Authors:** Lianne P. de Vries, Margot P. van de Weijer, Lannie Ligthart, Gonneke Willemsen, Conor V. Dolan, Dorret I. Boomsma, Bart M. L. Baselmans, Meike Bartels

**Affiliations:** 1 Department of Biological Psychology, Vrije Universiteit Amsterdam, Van der Boechorststraat 7, 1081 BT Amsterdam, The Netherlands; 2 Amsterdam Public Health Research Institute, Vrije Universiteit Amsterdam, Amsterdam, The Netherlands; 3 Neuroscience Amsterdam, Amsterdam, The Netherlands; 4 Institute for Molecular Bioscience, The University of Queensland, Brisbane, QLD Australia

**Keywords:** ASEBA, Questionnaires, Psychopathology, Internalizing problems, Externalizing problems, Attention problems, Classical twin design

## Abstract

The adult self report (ASR) is a well-validated instrument with multiple scales relating to adult psychopathology. Recently, an 18-item version has been introduced, the brief problem monitor (BPM) to measure Internalizing behavior (INT), Externalizing behavior (EXT), and attention problems (ATT). The present study compared the BPM and ASR and investigated how well the BPM can serve as a supplement or an alternative for the ASR for specific clinical and scientific purposes. In a large sample of adult twins (N = 9.835) from the Netherlands Twin Register (NTR), we compared the internal consistency, clinical classification concordance, means, and variances of the ASR and BPM. Using the classical twin design, we investigated the genetic covariance structure. For external validation, the associations between subjective well-being and different subscales of the ASR and BPM were compared. The internal consistency of the BPM scales (around α = 0.75) was somewhat lower than the ASR (α ~ 0.85). The BPM Externalizing scale showed the lowest internal consistency (α = 0.63). ASR and BPM scores showed good clinical classification concordance (0.61–0.80) and high correlations (r > 0.88). A small reversed sex difference in the BPM Externalizing scale appeared (women > men). Genetic (0.34–0.54) and environmental components (0.46–0.66) explained the variance to a similar extent for the ASR and BPM. The phenotypic and genetic associations with well-being were comparable. In situations where sum scores are sufficient, the BPM performs as well as the longer ASR. Depending on the situation and goal, it is worth considering the BPM as an alternative for the ASR to reduce the participant burden.

**Electronic supplementary material:**

The online version of this article (10.1007/s10519-020-10001-3) contains supplementary material, which is available to authorized users.

## Introduction

The adult self report (ASR) is a well-validated instrument to assess adult psychopathology and is used for clinical and research purposes in mental health, forensic, counseling, and medical settings. The ASR is part of the Achenbach System of Empirically Based Assessment (ASEBA) taxonomy and consists of items to assess adaptive functioning and problems (Achenbach and Rescorla 2003; Achenbach et al. 2017). The ASR comprises eight syndrome scales. The combination of the syndrome scales *Anxious/Depressed* (18 items), *Withdrawn* (9 items), and *Somatic Complaints* (12 items) results in the broadband scale Internalizing problems. The combination of the syndrome scales *Aggressive Behavior* (15 items), *Rule-breaking Behavior* (14 items), and *Intrusive Behavior* (6 items) forms the broadband scale Externalizing problems. The other syndrome scales are *Attention Problems* (15 items) and *Thought Problems* (10 items). *Other Problems* (21 items) include items that did not qualify for any syndrome. The remaining 11 items measure adaptive functioning and are not included in analyses. The total score on the ASR, based on all problem items (N = 120) represents the *Total Problems* score for adult psychopathology.

Recently, Achenbach and Ivanova developed the Brief Problem Monitor (BPM/18-59, here abbreviated as BPM) (Achenbach and Ivanova 2018) to fill the need for frequent brief assessments to evaluate responses to interventions and to monitor functioning. The BPM consist of 18 items from the ASR and Adult Behaviour Checklist (ABCL) (Achenbach and Rescorla 2003; Achenbach and Ivanova 2018) to measure Internalizing behavior, Externalizing behavior, and Attention Problems. In a large multicultural and adult sample (N = 11.790, from 17 societies), items for the BPM were selected that displayed the highest factor loadings on an Internalizing, Externalizing or Attention problems factor, and that discriminated best between participants referred for mental health services and healthy controls (Achenbach and Ivanova 2018). The BPM includes three scales, each assessed with 6 items: *Internalizing behavior* (INT), *Externalizing behavior* (EXT), and *Attention Problems* (ATT). The score based on all 18 items represents the *Total Problems* score (TOT).

### Comparison of the ASR and BPM

There is ample evidence that the ASEBA scales, including the ASR, have good psychometric properties, and are suitable to classify children and adults in the clinical or normal range of psychopathology (e.g. Achenbach and Rescorla, 2003; Bilenberg 1999; Rescorla and Achenbach 2004; Schmeck et al. 2001; Strömbäck et al. 2015; Zasepa and Wolanczyk 2011). The extensive number of subscales gives a complete overview of non-adaptive functioning in different problem areas. However, this extensiveness presents a burden for the participant. Generally, it takes 15–20 min to complete the ASR. Multiple meta-analyses have shown that long questionnaires (compared to short questionnaires) are associated with a lower response rate, lower accuracy, and lower compliance (Yammarino et al. 1991; Deutskens et al. 2004; Rolstad et al. 2011). Generally, the ideal and maximum survey length is considered to be 10 and 20 min, respectively (Revilla and Ochoa 2017). However, factors such as personalized contact and the survey content also have a bearing on this issue (Cook et al. 2000; Sheehan 2001).

The BPM is not designed as an alternative to the ASR as it lacks many scales, but in specific situations the BPM may be preferable to the ASR with respect to response rate, accuracy, compliance, and ease of administration. In view of its length, the BPM is more suitable for large scale (survey) studies and as a supplement for brief repeated assessments and frequent administration (monitoring). However, being much shorter, an important issue is the psychometric quality (reliability and validity) of the BPM.

### Goal of the Present Study

The aim of the present study was to compare the BPM and ASR in multiple ways. To avoid measurement error and possible inexplicable differences in item scores (i.e. due to different moments of assessment or other different circumstances), we used the ASR survey items to both compute the ASR scores (following the standard ASEBA manuals), as well as the BPM scores (by solely selecting the items that are part of the BPM). This inevitably also leads to the limitation that people could have responded differently if they would only have been presented with the BPM items. Given, though, that our study is important for the decision to use the ASR or BPM in future studies we decided to first conduct the study in this way to avoid potential harm to our longitudinal cohort studies due to survey switching. We compared the BPM and ASR scores with respect to internal consistency, means and variances, correlations with each other and with an external measure, and their genetic structure. We focused on the Internalizing (INT), Externalizing (EXT), Attention (ATT), and Total (TOT) problem scales of the BPM and ASR. We assessed the internal consistency of the scales using Cronbach’s alpha, compared the means and variances, and calculated correlations between the ASR, BPM, and subjective well-being. Subjective well-being (SWB) was measured using the Satisfaction with Life scale (Diener et al. 1985). SWB is used as an external criterion to compare the ASR and BPM, since subjective well-being and psychopathology show a strong negative relation. Using the ASR scores, Baselmans et al. (2018) found a negative relation with subjective well-being in adults. Comparable correlations between SWB and the BPM scores provide evidence for the BPM validity. Next, we compared diagnostic use of the scales. Ideally, the same people are classified as scoring in the clinical range when administering the ASR or BPM. To investigate the genetic structure, and compare the sources of individual differences in the ASR and BPM, we applied the classical twin design. As an external validation, we compared the associations of the ASR and BPM with SWB in bivariate twin models.

## Method

### Participants

We analyzed data of participants from the Netherlands Twin Registry (NTR), established by the Department of Biological Psychology at the VU University in Amsterdam (Boomsma et al. 2002b; Willemsen et al. 2013). The NTR sample is a population-wide non-clinical sample of twins and their family members in the Netherlands. Adolescent and adult twin were first recruited through city councils. The recruitment of participants is ongoing and nowadays means of recruitment are the yearly NTR newsletter *Twinfo*, the NTR social media and website and national events (Ligthart et al. 2019). The NTR has registered about 29% of all Dutch twin-pairs born between 1970 and 1981. For other birth cohorts, coverage is considerably lower due to lack of systematic recruitment. The NTR collects longitudinal survey data about lifestyle, personality, and psychopathology every 2/3 years in adolescent and adult twins and their families. The current study uses data on psychopathology collected in survey 8 in adults between 2009 and 2012 (Willemsen et al. 2013).

From the total sample of adult twin and family members, we selected adult participants who were part of a twin pair and had less than 8 missing items on the ASR (N = 10.019). Twins with unknown zygosity (N = 184) were excluded, leaving a final sample of 9.835 participants (mean age: 31.0, *SD* = 14.5, 68.1% females). The sample included 3.255 complete twin pairs (N = 6.510) and 3.325 incomplete twin pairs. The sample included 1.344 monozygotic male (MZM), 822 dizygotic male (DZM), 3.436 monozygotic female (MZF), 1.815 dizygotic female (DZF) and 2.418 dizygotic opposite-sex (DOS) twins (from complete and incomplete twin pairs).

Zygosity was determined by DNA typing in 53% of the same-sex twin pairs. For the other same-sex twin pairs, zygosity was based on eight items on physical similarity and the frequency of confusion of the twins by parents, family members, and strangers (Willemsen et al. 2005). Agreement between zygosity assignment based on questionnaire information and zygosity determined by DNA markers is around 93%.

### Measures

#### The Adult Self Report

The ASR is part of the ASEBA taxonomy (Achenbach and Rescorla 2003). The ASR consists of 120 problem items, which are distributed over the syndrome scales as mentioned before. The ASR items can be combined to create several DSM-oriented problem scales (e.g. depression, anxiety), and a substance use scale.

In completing the ASR, participants report their behavior, thoughts, and feelings of the previous 6 months by rating how applicable the items are. Each item is rated from *0* = *not true, 1* = *somewhat true,* to *2* = *very true*. Summing the score on all problem items results in a Total problems score, ranging from 0 to 240. Two example items are *“I do not get along with other people”* and *“I drink too much alcohol or get drunk”.*

As a result of missing data, scores based on all items per ASR scale were not always available for each participant. To increase the number of data points, we imputed missing data. The total scale score was only computed (and missing data imputed) when less than 8 items of the complete scale were missing, in line with Achenbach and Rescorla (2003). For the subscales, if at least 80% of the items of the subscale was completed, missing data were imputed and a scale score constructed. Data were imputed by computing the average of the existing scores of the subscale and replacing missing values with this scale average. For the ASR, the percentage of participants for which we imputed at least one item score was 21.5% for the Total scale, 14.2% for the Internalizing scale, 13.2% for the Externalizing scale and 9.0% for the Attention scale.

#### The Brief Problem Monitor

The BPM is an 18-item questionnaire, comprising a selection of ASR items (Achenbach and Ivanova 2018). The BPM includes three scales; Internalizing (INT), Externalizing (EXT), Attention problems (ATT). The sum of all 18 item scores provides the Total problems (TOT) score. Participants report their behavior, thoughts, and feelings of the previous 6 months by rating how applicable the items are. Each item is rated from *0* = *not true, 1* = *somewhat true,* to *2* = *very true*. Two example item are *“I lack self-confidence”* and *“I fail to finish things I should do”.*

We computed the BPM scores by summing the scores on the appropriate ASR items in the data set. We imputed missing scores in the same way as we did with the ASR items (see above). The percentage of participants for which we imputed at least one item score in the BPM was 8.9% for the Total scale, 2.3% for the Internalizing scale, 3.3% for the Externalizing scale and 3.3% for the Attention scale.

#### Subjective Well-Being

Subjective well-being (SWB) was assessed with the Satisfaction with Life scale (SWLS) (Diener et al. 1985). The scale has five items with a 7-point Likert scale, ranging from *1* = *strongly disagree* to *7* = *strongly agree*. The SWLS shows high internal consistency and high temporal reliability (2-month test–retest: 0.82, and coefficient alpha was 0.87) (Diener et al. 1985). The internal consistency of the SWLS in our sample is similar, with a Cronbach’s alpha of 0.876. An example question is ‘*In most ways my life is close to ideal’*.

### Analyses

#### Internal Consistency and Descriptive Statistics

We first assessed the internal consistency of the ASR and BPM scales using Cronbach’s alpha. Next, we compared the means and variances of the scales, and calculated phenotypic correlations between the ASR, BPM, and subjective well-being scores in R (R Core Team 2017). To correct the correlation for the overlap in items, we also computed the correlations between the score of the ASR items when excluding the BPM items and the BPM score. As an extra check, we correlated the ASR and BPM scores across two time points. First, we correlated the ASR score with the ASR score 4 years later (ASR 2) and the BPM with the BPM score 4 years later (BPM 2). These correlations reflect the temporal stability of the ASR and BPM scores. Next, we correlated the score of the BPM at time point 1 and ASR at time point 2 and vice versa, the ASR score at time point 1 and the BPM score at time point 2. If these correlations are similar to the temporal correlations, this indicates high similarity between the ASR and BPM scales. In the rest of the analyses, we used the data of wave 1. The data of wave 2 were only used to compute the temporal stability.

#### Clinical Classification Concordance

The ASR is frequently used as a screening tool to classify individuals in the normal or clinical range of psychopathology. To assess the agreement of the ASR and BPM in clinical classification, we ran a concordance analysis (Landis and Koch 1977; Kwiecien et al. 2011). We converted raw scores to age and gender norm-based T-scores for all ASR and BPM subscales (based on sample specific means and standard deviations for 4 groups based on gender (males/females) and age (18–35 and > 35)) (Achenbach and Rescorla 2003). Participants scoring above 63 on the ASR scales are considered to be in the clinical range (Achenbach and Rescorla 2003). Similarly, participants scoring above 64 on the BPM scales are at high risk for psychopathology, according to Achenbach and Ivanova (2018). Using a binary variable, we scored those passing those thresholds as 1 and people with scores lower than the threshold as zero. Using Cohen’s kappa (Cohen 1960; Landis and Koch 1977), we tested the classification concordance based on the ASR and BPM (sub)scales.

#### Twin Modelling

Monozygotic (MZ) twin pairs share (nearly) all genes, whereas dizygotic (DZ) twin pairs share on average half of their segregating genes. Based on this difference in genetic relatedness, the classical twin design can be used to decompose the observed or phenotypic variance of traits into genetic and environmental variance components (Boomsma et al. 2002a). Additive genetic variance (A) represents the additive variance explained by all alleles that influence the phenotype. It is called additive because the effect of each allele is based on a linear model, in which the phenotypic scores are linearly related to each genotype. Non-additive genetic variance (D) arises due to interactions between alleles at the same locus (dominance) or between alleles at different loci (epistasis). The dominance variance is the genetic variance that is not explained by the linear regression of the phenotypic scores on the genotype. The environmental variance consists of a common environmental variance component (C) (variance shared by family members) and a non-shared environmental component (E) (part of the variance that is unique for an individual). In the classical twin design, the effects of C and D cannot be estimated simultaneously, therefore a choice for an ADE or ACE model is made based on the pattern of twin correlations. An ADE model is appropriate if twice the DZ correlation (r_DZ_) is smaller than the MZ correlation (r_MZ_), 2*r_DZ_ < r_MZ_.

The ASR and BPM scores were strongly skewed right, with most participants scoring low on the ASR and BPM. This non-normality may bias estimates of variance components based on the classical twin design (Derks et al. 2004). To prevent biased estimates, we transformed the data into categorical data with three groups (low, middle and high). The groups were created and thresholds determined by creating three groups of equal sizes (33%). We applied the liability threshold model on the created ordinal ASR and BPM variables (Neale et al. 1994). The liability threshold model assumes a normally distributed liability underlying the psychopathology. People differ in their lability for psychopathology (e.g. internalizing problems or attention problems) with most individuals showing a low liability and vulnerability for psychopathology and few scoring higher. We chose to include two thresholds, beyond which individuals will fall in the middle or high psychopathology group. The ASR and BPM scores were thus analyzed as ordinal variables*.* In the two-threshold model the liability variance can be decomposed into genetic and environmental components. The prevalence of the low, middle, and high scores is expressed by means of the thresholds.

Although we included adult participants from all ages in the analyses (max age is 97), we did not include age in the twin models. Prior analyses in the NTR sample have shown that the genetic architecture on psychopathology scales is stable in adulthood (Kan et al. 2013; Nivard et al. 2015). Furthermore, as can be seen in Supplementary Table S1, the correlations of age with the ASR and BPM scales were comparable and small (around − 0.10 in all scales), with overlapping confidence intervals for each subscale, except for the Externalizing scale.

For every ASR and BPM scale, the assumptions of equal thresholds across twin order and zygosity were checked in a saturated model. Although a few scales did show a deterioration of the model fit when equating the thresholds across twin order and zygosity, we chose to equate them in further models, as the differences were small and not clinically relevant. Since it is known that men and women typically differ on their scores on the problems scales [women score higher on the Internalizing and Attention scales and men score higher on the Externalizing scale; (Achenbach and Rescorla 2003; Kan et al. 2013; Baselmans et al. 2018)], we allowed for sex differences in the thresholds. Twin correlations were estimated based on the saturated model.

Next, using univariate twin models, we estimated genetic and environmental contributions to the phenotypic variance of the ASR and BPM scales by decomposing the phenotypic variance into A, C (or D), and E variance components. Using the log-likelihood ratio test, the full ACE/ADE models were compared to nested submodels to test the significance of parameter estimates. To investigate whether the variance components A and C or D significantly contributed to the total variance and/or covariance, we tested whether constraining them resulted in a significant deterioration of model fit. The fit of the different models (both for the saturated as well as the genetic models) was compared by means of the log-likelihood ratio test (LRT). The difference in minus two times the log-likelihood (-2LL) between two nested models has a χ2 distribution with the degrees of freedom (df) equaling the difference in df between the two models. If a p value from the χ2 -test was higher than 0.01, the fit of the constrained model is not significantly worse than the fit of the more complex model. However, as the classical twin design is known to lack power to detect non-additive genetic effects (Posthuma and Boomsma 2000), we will show both the results of the full ACE or ADE model and the results of the best fitting model. In addition, 95% confidence intervals were calculated for the parameter estimates of the full model and the best fitting model. All analyses were performed with OpenMx (Boker et al. 2011) in R.

Next, we ran bivariate twin models to compute the overlap of the ASR and BPM scores. When investigating multiple traits we can obtain genetic and environmental correlations between the phenotypes, in addition to the phenotype specific variance and covariance decomposition. The genetic correlations reflect the overlap between the genetic factors influencing the ASR and BPM scores.

#### External Validation

As an external validation, we compared the relation of the ASR and BPM with subjective well-being. First, we performed a regression to compare the prediction for well-being based on the ASR and BPM scores. Since the data includes related individuals the regression was performed with a generalized estimation equation with robust standard errors to correct for the presence of related individuals (Minică et al. 2015).

Next, we ran bivariate twin models to compare the overlap of the ASR scores and subjective well-being to the overlap of the BPM and subjective well-being. We estimated genetic and environmental contributions to the bivariate phenotypic covariance matrix by decomposing the phenotypic covariance matrix into (2 × 2) A, C (or D), E covariance matrices. Furthermore, we calculated the genetic and environmental correlations between the phenotypes. The genetic correlations reflect the overlap between the genetic factors influencing the phenotypic traits of psychopathy and well-being.

## Results

### Internal Consistency

The Cronbach’s alpha’s, reflecting the internal consistency, of the ASR scales were α = 0.95 (Total), α = 0.91 (Internalizing), α = 0.84 (Externalizing) and α = 0.81 (Attention problems). The Cronbach’s alpha’s of the BPM scales Total (α = 0.86), Internalizing (α = 0.79), and Attention (α = 0.71) were lower, but still good. This is to be expected as the subscales are based on only 6 items. Only the Externalizing BPM scale shows a lower Cronbach’s alpha of 0.63.

### Descriptives

The phenotypic correlations between the whole ASR and BPM scores are 0.916 (95% CI 0.912−0.920) for Total, 0.879 (95% CI 0.874−0.885) for Internalizing, 0.833 (95% CI 0.826−0.840) for Externalizing, and 0.899 (95% CI 0.895−0.904) for the Attention problems scales (see Table 1). The correlations between the score of the ASR items when excluding the BPM items and the BPM scores are still relatively high, but lower than the part-whole correlations. As an extra check, we correlated the ASR and BPM scores across two time points. First, the correlation between the ASR scores at two time points (ASR 1 and ASR 2) and the BPM scores at two time points (BPM 1 and BPM 2) showed temporal stability for all scales (r ~ 0.7). The correlations across time between the scales score are somewhat lower, but there are some overlapping confidence intervals with the temporal correlations (see Table 1). This indicates a high similarity between the ASR and BPM.

**Table 1:** Correlations between the ASR and BPM scales at two different time points (95% CI)

	Correlation	TOT	INT	EXT	ATT
	ASR–BPM	0.916 (0.912–0.920)	0.879 (0.874–0.885)	0.833 (0.826–0.840)	0.899 (0.895–0.904)
	ASR _excluding BPM items_–BPM	0.863 (0.856–0.869)	0.802 (0.793–0.810)	0.703 (0.690–0.715)	0.677 (0.664–0.689)
Temporal stability	ASR 1–ASR 2	0.739 (0.724–0.753)	0.716 (0.700–0.732)	0.667 (0.649–0.684)	0.707 (0.691–0.723)
	BPM 1–BPM 2	0.731 (0.715–0.749)	0.664 (0.646–0.681)	0.721 (0.705–0.736)	0.631 (0.611–0.649)
Cross-time and cross-scale	BPM 1–ASR 2	0.695 (0.678–0.711)	0.653 (0.634–0.670)	0.569 (0.548–0.590)	0.636 (0.617–0.654)
	ASR 1–BPM 2	0.699 (0.681–0.715)	0.639 (0.619–657)	0.654 (0.635–0.672)	0.619 (0.599–0.638)

The ASR 2 and BPM 2 scores are scores from 4 years later

For all descriptives, see Supplementary Table S2. Paired t-tests’ show that the weighted means (divided by the number of items) of the Total (*diff* =  − 0.051, *t* = 33.23, *p* < 0.001, *d* = 0.39), Internalizing (*diff* =  − 0.018, t = 8.42, *p* < 0.001, *d* = 0.10), Externalizing (*diff* =  − 0.078, *t* = 38.31, *p* < 0.001, *d* = 0.45) and Attention scale (*diff* =  − 0.022, *t* = 11.78, *p* < 0.001, *d* = 0.14) of the ASR are significantly lower than the weighted means of the comparable BPM scales. The differences in the Internalizing and Attention scale are small (d < 0.20, (Cohen 1988)), whereas the differences of the Total and Externalizing scale can be considered moderate (d = 0.39 and d = 0.45).

**Fig. 1:**
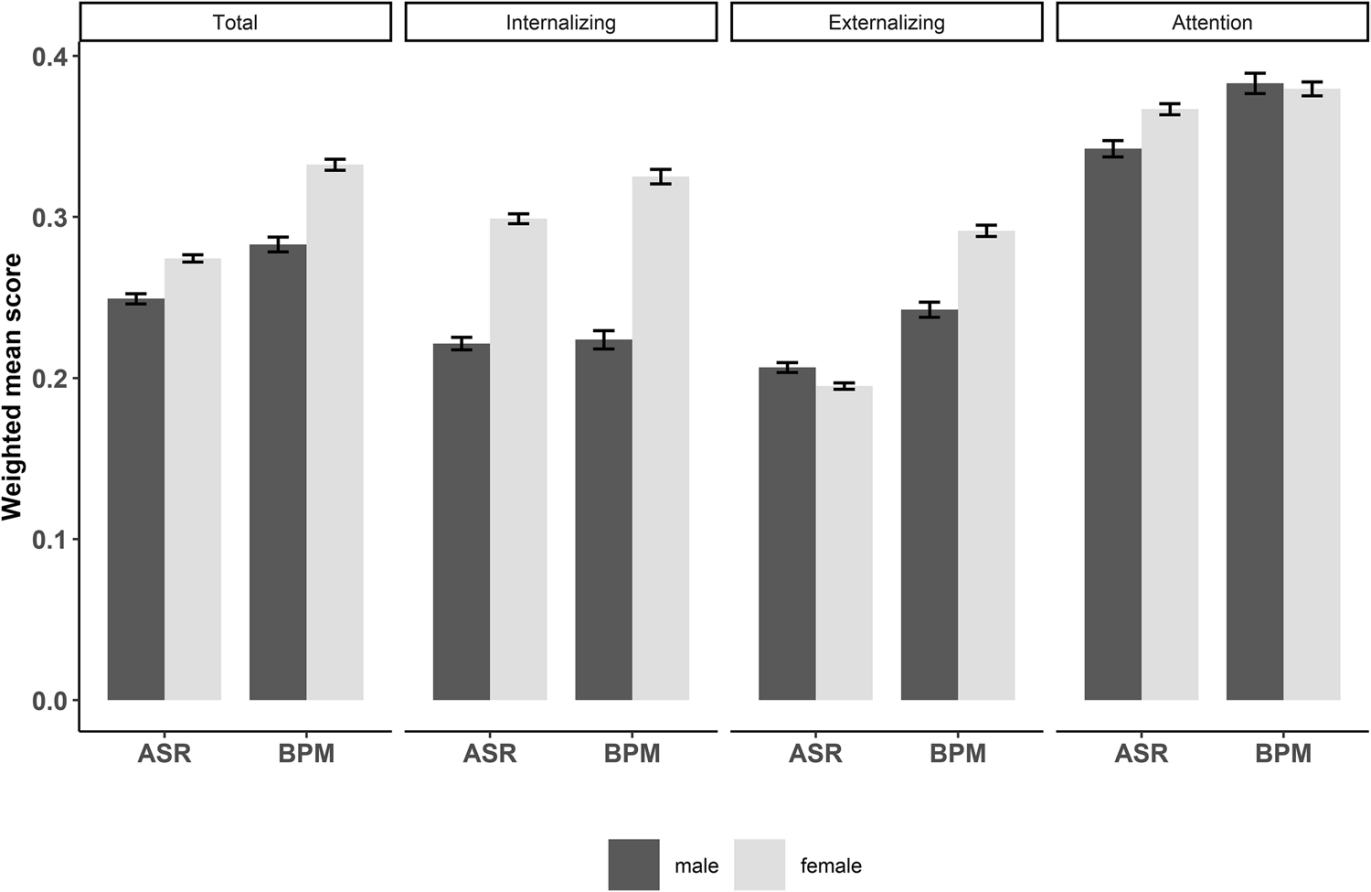
The weighted means of the ASR and BPM subscales, compared for men and women. All sex differences, except in the Attention BPM scale, are significant (p < 0.001)

The weighted means of the ASR and BPM are shown in Fig. 1, separately for men and women (see Supplementary Table S2 for the statistics). The expected sex differences were observed in the ASR scores. Women scored higher on the Total, Attention, and Internalizing subscale, whereas men scored higher on the Externalizing subscale. For the BPM, women scored higher on the Total and Internalizing scale as well, whereas no sex-difference is observed for Attention problems. Unexpectedly, women scored higher on the BPM Externalizing subscale than men. For SWB, men (*M* = 27.51*, SD* = 5.11) report somewhat higher levels than women (*M* = 27.07, *SD* = 5.56), resulting in a small (d = 0.08) but significant sex difference. This is in line with previous studies. Batz and Tay (2018) summarized the literature on sex differences in well-being and found that in the majority of the studies to life satisfaction men have higher levels of life satisfaction than women, although the size of the sex difference is small and this small sex difference might not be relevant.

### Clinical Classifications

To test the concordance of the ASR and BPM in categorizing people in the normal or clinical range, we compared the proportion of participants that exceeded the clinical cut off score (T-score > 63 (ASR) or 64 (BPM)) of the subscales. The proportions of participants classified as having clinical problems are similar for the scales (overall *M* = 10.7%). The scales showed good clinical concordance according to the Kappa score guidelines (Landis and Koch 1977) (Total = 0.736, Internalizing = 0.674, Externalizing = 0.625 and Attention = 0.690). Also, when dividing the sample in four groups based on gender and age (18–35 and > 35 years), high Kappa scores and no differences between the ASR and BPM in proportions of individuals over the clinical cut-off were observed (see Supplementary Table S3).

### Genetic Analyses

We estimated the twin correlations and cross twin-cross trait correlations for the ASR and BPM scales in a saturated model. As expected, constraining the thresholds to be equal for males and females resulted in a significant deterioration of model fit, except in the Attention problem scales (see Supplementary Table S4).

The ASR and BPM scales show comparable twin correlations with overlapping confidence intervals (except the Total scales), indicating that the ASR and BPM scores are heritable to the same extent (see Table 2). Furthermore, cross-twin cross-trait correlations are higher for MZ than for DZ twins. The MZ correlations were greater than twice the DZ correlations, indicating a contribution of dominant genetic effects (D) besides additive genetic effects. Therefore, we proceeded with ADE models.

**Table 2:** Twin correlations and cross twin-cross trait correlations for the ASR and BPM scales (95% CI: ± 1.96*SE)

		MZ		DZ	
		ASR	BPM	ASR	BPM
TOT	ASR	0.541 (0.49, 0.59)		0.239 (0.16, 0.31)	
	BPM	0.467 (0.42, 0.51)	0.428 (0.36, 0.49)	0.203 (0.14, 0.26)	0.158 (0.07, 0.25)
INT	ASR	0.465 (0.40, 0.52)		0.224 (0.14, 0.30)	
	BPM	0.420 (0.37, 0.46)	0.463 (0.41, 0.52)	0.163 (0.10, 0.22)	0.157 (0.08, 0.24)
EXT	ASR	0.444 (0.38, 0.51)		0.196 (0.11, 0.28)	
	BPM	0.383 (0.34, 0.43)	0.343 (0.25, 0.43)	0.109 (0.05, 0.17)	0.098 (− 0.02, 0.21)
ATT	ASR	0.455 (0.39, 0.52)		0.161 (0.08, 0.24)	
	BPM	0.414 (.37, 0.46)	0.402 (0.32, 0.48)	0.205 (0.14, 0.26)	0.106 (0.01, 0.21)

**Fig. 2:**
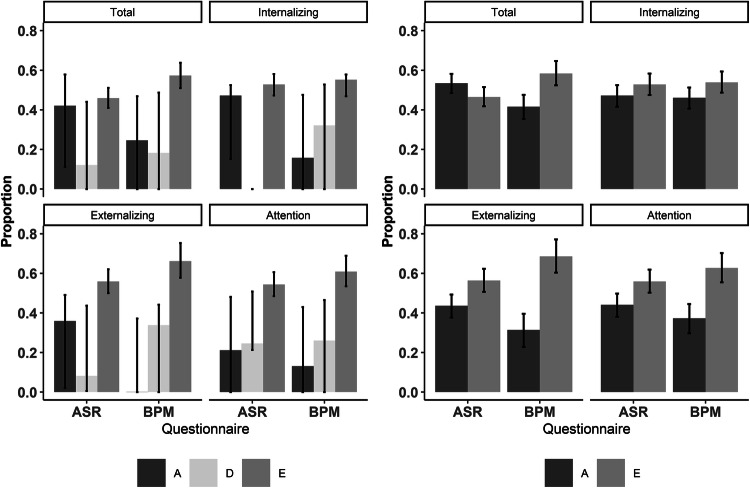
Standardized estimates (95% CI) for the additive genetic, non-additive genetic and non-shared environmental influences on the ASR and BPM in the ADE and AE model

Figure 2 shows the results of the full univariate ADE models and the best fitting models, AE models (see Supplementary Table S5 for the model fitting results). Genetic effects explained 44–54% of the phenotypic variation in the ASR scales, while for the BPM genetic estimates were between 34 and 48%. The remaining variance is explained by non-shared environmental effects. All confidence intervals (in both the ADE and AE models) of the comparable ASR and BPM scales overlap, indicating a similar genetic architecture for the ASR and BPM (see Fig. 2 and Supplementary Table S6).

The best fitting bivariate model, including the scales of the ASR and BPM are AE models. These models show high genetic and environmental correlations between the ASR and BPM. The genetic correlations for the Total (0.952 [95% CI 0.93−0.98]), Internalizing (0.928 [95% CI 0.89−0.95]), Externalizing (0.871 [95% CI 0.80−0.94]) and Attention problems scale (0.944 [95% CI 0.90−0.99]) are close to unity, indicating that the additive genetic influences might be the same for the ASR and BPM scores. Similarly, the environmental correlations are high for the Total (0.844 [95% CI 0.83 − 0.85]), Internalizing (0.807 [95% CI 0.77−0.83]), Externalizing (0.759 [95% CI 0.71−0.80]) and Attention problems scale (0.847 [95% CI 0.84−0.88]).

### External Validation

**Fig. 3:**
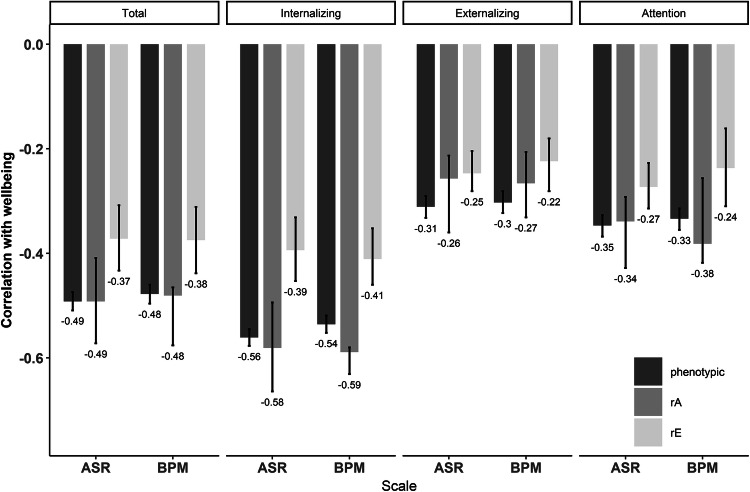
The phenotypic, genetic and environmental correlations (95% CI) for the overlap between the subjective well-being and ASR scores and the subjective well-being and BPM scores

Phenotypically, subjective well-being (SWB) is negatively correlated to the ASR and BPM scores, with the strongest correlations for the Internalizing subscales (see Fig. 3 and Supplementary Table S7). The ASR and BPM predict SWB to the same extent, with overlapping confidence intervals according to the regressions. For example, for the Total scales, the ASR estimate is − 0.499 (95% CI − 0.525, − 0.473) and the BPM estimate − 0.485 (95% CI − 0.512, − 0.459) (see Supplementary Table S8).

The bivariate analyses show that the additive genetic influences on the association of the ASR scores and SWB ranges from 0.41 to 0.52. For the BPM, the estimated contribution of additive genetic effects to the association with SWB is similar, ranging from 0.39 to 0.50. The remaining covariance is accounted for by non-shared environmental influences. All confidence intervals overlap, indicating that the associations of the ASR and BPM with SWB can be explained in the same way (see Supplementary Tables S9, S10 and S11 for the assumption checking and model fitting results).

Furthermore, the genetic and environmental correlations between SWB and the ASR and SWB and the BPM scores were similar (see Fig. 3 and Supplementary Table S7). This indicates an overlap in the genetic and environmental influences on subjective well-being and the ASR and BPM scores.

## Discussion

The present study aimed to investigate how well the BPM can serve as an alternative for the ASR for clinical and scientific purposes. In a large sample of twins from the Netherlands Twin Register (NTR) we compared the ASR and BPM scores in multiple ways. We investigated the internal consistency, means, concordance in clinical classifications, the underlying variance structure, and association with subjective well-being. In summary, the results showed higher (weighted) mean scores on the BPM compared to the ASR, but strong correlations between them. The concordance in classifying people in the clinical range between the scales was high. The twin modelling showed a similar underlying genetic architecture for the ASR and BPM and high genetic and environmental correlations. Furthermore, bivariate models showed that the association of both scales with subjective well-being can be explained by genetic and environmental components to the same extent.

The high correlations between the scores provides support for the idea that the BPM measures the same construct as the ASR, namely psychopathology. The higher weighted means of the BPM compared to the ASR indicate that the items selected for the BPM reflect more common rated problems compared to the rest of the ASR items. This suggests that the selection process of Achenbach and Ivanova (2018) was effective. As a result, problems can be detected more efficiently with the BPM compared to the ASR.

We observed an unexpected sex difference for Externalizing problems in the BPM. As found in previous research, we showed that men scored higher on the Externalizing scale of the ASR, whereas women scored higher on Internalizing problems. In contrast, for the BPM, women scored higher on both the Externalizing and Internalizing problems scale. We did notice that the content of the BPM Externalizing items applies more to women than men in our sample. On four of the six items selected for the BPM, females scored significantly higher than males. For example, the items “*I get upset too easily*” (*M*_female_ = 0.327 vs *M*_male_ = 0.142) and “*My behavior is very changeable*” (*M*_female_ = 0.264 vs *M*_male_ = 0.212) are two of the six items. Also in general, women are found to score higher on those emotionality related traits and mood instability than men (Lee and Ashton 2004; Ashton et al. 2006). The more physical and aggressive items (e.g. “*I get into many fights*” (*M*_male_ = 0.018 vs *M*_female_ = 0.011) or “*I drink too much alcohol or get drunk*” (*M*_male_ = 0.311 vs *M*_female_ = 0.161)) that apply more to men than women in our sample and in general (Archer 2004; Schober et al. 2009) have not been selected for the BPM.

The concordance between the scales in clinical classification can be considered high (kappa > 0.60). As described, the ability of the ASR scale to discriminate between referred adults (adults who received mental health or substance abuse services) and controls is good. The high concordance indicates that the BPM can also be used to quickly (re-)classify people on psychopathology.

Further evidence for the similarity between the ASR and BPM is found in the high phenotypic correlations between the scores and the similar underlying genetic architecture. Genetic and environmental effects both explain about half of the variance in the ASR and BPM scales which is comparable to the heritability of traits in general (Polderman et al. 2015). The estimate of the genetic effect is somewhat lower in the BPM scales than in the ASR scales. An explanation for this lower genetic and therefore higher environmental effect might be the increase in measurement error, as the BPM is based on only 18 items and the ASR on 120 items. This suggests a lower reliability of the BPM than the ASR. However, the differences in estimates were small and the confidence intervals did overlap substantially. The genetic and environmental correlations between the scales are close to unity, indicating the same genetic and environmental influences on the ASR and BPM scores.

As an external validation, we found that the ASR and BPM predict subjective well-being to the same extent. In addition, the bivariate analyses showed that the covariance structure between well-being and both scales is comparable and the genetic and environmental correlations between well-being and the ASR and BPM scales are similar. This indicates that overlapping genetic and environmental influences both increase feelings of subjective well-being and decrease psychopathology, either measured with the ASR or BPM.

All above findings suggest that the ASR and BPM assess liability to psychopathology in the same way. Therefore, the BPM can be considered as an alternative for the ASR in some scientific and clinical circumstances. However, depending on the specific use of the questionnaire scores and goal, there are a few limitations in using the BPM instead of the ASR. First, when selecting 18 items from 120 items, by definition, a lot of information is lost. Whereas the ASR broadband scales Internalizing and Externalizing each consist of three subscales (respectively *Anxious-Depressed, Withdrawn* and *Somatic Complaints* and *Aggression, Rule-Breaking Behavior* and *Intrusive behavior*), the BPM Internalizing and Externalizing scale consist of a few items from these subscales. Therefore, only the broad category is measured and variance is lost.

The results indicate that the BPM Internalizing, Attention, and Total problem scales measure psychopathology relatively well (a high internal consistency, correlation and resemblance to the ASR). The results of the Externalizing problems scale indicate a somewhat poorer performance. The internal consistency is lower (α = 0.63) and the sex difference is opposite as expected, with higher scores for women. If replicated, the lower performance of the Externalizing scale is an area for improvement. When checking the items, a few ASR items with high variance are not included in the BPM. Potential items for the Externalizing scale could be “*I am stubborn, sullen or irritable*”, or “*I argue a lot*”. Based on face validity, including some of the more physical items, like “*I get into many fights*” might increase the reliability and performance of the Externalizing scale. If this reversed sex effect in the BPM Externalizing scale replicates in other samples and studies, we recommend recreating the Externalizing subscale of the BPM to be in line with the other ASEBA Externalizing scales. Otherwise, this subscale should be used with caution.

Additionally, the structure is one of the strengths of the ASR. The items are not ordered, thereby randomizing and limiting the clustering of items of the same problem area. The addition of the 11 positive items decreases the focus on specific problems even further. The BPM has only 18 items, creating somewhat larger clusters of problem items. This may impact the completion of the questionnaire, by making the participants more aware of what is measured.

As already noted in the introduction, a limitation of the current study is the BPM data collection. We used a data set with ASR scores and computed BPM scores ourselves based on the answers to the selected ASR items. Participants did not actually complete the BPM. The format and ordering of a questionnaire influences completion (e.g. McFarland et al. 2002). Presenting items randomized or grouped can affect faking and the reliability and validity. Therefore, people could respond differently when only presented with the BPM items instead of all ASR items. However, using a subset (the 18 BPM items) of the ASR data to compare the scales can be seen as both a strength and a weakness of the study. The data are not completely independent and this may lead to more false positives in our results. However, the correlations between the BPM scores and ASR scores without the BPM items included are still high. In addition, correlating the BPM scores to the ASR scores in the same sample a few years later indicates a high correlation as well. Furthermore, cross twin-cross trait correlations are similar to the ASR and BPM (same trait) twin correlations, indicating reliable correlations. In addition, the strength of this sample is the avoiding of non-equal samples. Since the comparison is based on the same sample, the results are not affected by any other characteristic of the samples. However, replication of our results is needed using different surveys for the ASR and BPM data collection instead of using the ASR survey items to compute both the ASR and BPM scores.

## Recommendations and conclusion

If our results are replicated, we propose that the BPM can serve as an efficient supplement or alternative for the longer ASR. In situations and research where only a sum score for problem behavior is necessary (e.g. genome-wide association studies), the BPM seems appropriate and may even be preferred to reduce the burden for participants. Furthermore, in clinical situations where problems have to be monitored over time, the BPM is more convenient and efficient. As mentioned, the BPM was designed to fill this need for frequent brief assessments and Achenbach and Ivanova (2018) stress that the BPM should be used in addition to the ASR. A recent trend is a decline in the use of sum and overall scores and increase in the use of network analyses. A longer questionnaire with more items, like the ASR is preferred for such analyses. Therefore, in research where a lot of information is needed or in diagnostic situations, the ASR is still preferred. In conclusion, depending on the situation and the goal, it is worth considering the BPM as an alternative for the ASR.

## Electronic supplementary material

Below is the link to the electronic supplementary material.

Supplementary file1 (DOCX 66 kb)

## References

[CR1] Achenbach TM, Ivanova MY (2018) Brief Problem Monitor ^TM^ for Ages 18–59 ( BPM / 18–59 ). University of Vermont, Research Center for Children, Youth, & Families, Burlington, VT

[CR2] Achenbach TM, Ivanova MY, Rescorla LA (2017). Empirically based assessment and taxonomy of psychopathology for ages 1½–90+ years: developmental, multi-informant, and multicultural findings. Compr Psychiatry.

[CR3] Achenbach TM, Rescorla LA (2003) Manual for the ASEBA adult forms & profiles. University of Vermont, Research Center for Children, Youth, & Families., Burlington, VT

[CR4] Archer J (2004). Sex differences in aggression in real-world settings: a meta-analytic review. Rev Gen Psychol.

[CR5] Ashton MC, Lee K, de Vries RE (2006). The HEXACO model of personality structure and indigenous lexical personality dimensions in Italian, Dutch, and English. J Res Pers.

[CR6] Baselmans BML, Willems YE, van Beijsterveldt CEM (2018). Unraveling the genetic and environmental relationship between well-being and depressive symptoms throughout the lifespan. Front Psychiatry.

[CR100] Batz C, Tay L, Diener E, Oishi S, Tay L (2018). Gender differences in subjective well-being. Handbook of well-being.

[CR7] Bilenberg N (1999). The child behavior checklist (CBCL) and related material: standardization and validation in Danish population based and clinically based samples. Acta Psychiatr Scand.

[CR8] Boker S, Neale M, Maes H (2011). OpenMx: an open source extended structural equation modeling framework. Psychometrika.

[CR9] Boomsma DI, Busjahn A, Peltonen L (2002). Classical twin studies and beyond. Nat Rev Genet.

[CR10] Boomsma DI, Vink JM, Van Beijsterveldt CEM (2002). Netherlands twin register: a focus on longitudinal research. Twin Res Hum Genet.

[CR11] Cohen J (1960). A coefficient of agreement for nominal scales. Educ Psychol Meas.

[CR12] Cohen J (1988). Statistical power analysis for the behavioural science.

[CR13] Cook C, Heath F, Thompson RL (2000). A meta-analysis of response rates in Web- or internet-based surveys. Educ Psychol Meas.

[CR14] Derks EM, Dolan CV, Boomsma DI (2004). Effects of censoring on parameter estimates and power in genetic modeling. Twin Res.

[CR15] Deutskens E, De Ruyter K, Wetzels M, Oosterveld P (2004). Response rate and response quality of internet-based surveys: an experimental study. Mark Lett.

[CR16] Diener E, Emmons RA, Larsen RJ, Griffin S (1985). The satisfaction with life scale. J Pers Assess.

[CR17] Kan KJ, Dolan CV, Nivard MG (2013). Genetic and environmental stability in attention problems across the lifespan: evidence from the netherlands twin register. J Am Acad Child Adolesc Psychiatry.

[CR18] Kwiecien R, Kopp-Schneider A, Blettner M (2011). Concordance analysis: part 16 of a series on evaluation of scientific publications. Dtsch Arztebl.

[CR19] Landis JR, Koch GG (1977). The measurement of observer agreement for categorical data. Biometrics.

[CR20] Lee K, Ashton MC (2004). Psychometric properties of the HEXACO personality inventory. Multivariate Behav Res.

[CR21] Ligthart L, van Beijsterveldt CEM, Kevenaar ST (2019). The Netherlands twin register: longitudinal research based on twin and twin-family designs. Twin Res Hum Genet.

[CR22] McFarland LA, Ryan AM, Ellis A (2002). Item placement on a personality measure: effects on faking behavior and test measurement properties. J Pers Assess.

[CR23] Minică CC, Dolan CV, Kampert MMD (2015). Sandwich corrected standard errors in family-based genome-wide association studies. Eur J Hum Genet.

[CR24] Neale MC, Eaves LJ, Kendler KS (1994). The power of the classical twin study to resolve variation in threshold traits. Behav Genet.

[CR25] Nivard MG, Dolan CV, Kendler KS (2015). Stability in symptoms of anxiety and depression as a function of genotype and environment: a longitudinal twin study from ages 3 to 63 years. Psychol Med.

[CR26] Polderman TJC, Benyamin B, De Leeuw CA (2015). Meta-analysis of the heritability of human traits based on fifty years of twin studies. Nat Genet.

[CR27] Posthuma D, Boomsma DI (2000). A note on the statistical power in extended twin designs. Behav Genet.

[CR28] R Core Team (2017) R: a language and environment for statistical computing. R Found. Stat. Comput. Vienna

[CR29] Rescorla LA, Achenbach TM, Maruish ME (2004). The achenbach system of empirically based assessment (ASEBA) for ages 18 to 90 years. The use of psychological testing for treatment planning and outcomes assessment: instruments for adults.

[CR30] Revilla M, Ochoa C (2017). Ideal and maximum length for a web survey. Int J Mark Res.

[CR31] Rolstad S, Adler J, Rydén A (2011). Response burden and questionnaire length: is shorter better? A review and meta-analysis. Value Heal.

[CR32] Schmeck K, Poustka F, Döpfner M (2001). Discriminant validity of the child behaviour checklist CBCL-4/18 in German samples. Eur Child Adolesc Psychiatry.

[CR33] Schober G, Björkqvist K, Somppi S (2009). Identifying a new subcategory of aggression: sex differences in direct non-verbal aggression. J Aggress Confl Peace Res.

[CR34] Sheehan KB (2001). E-mail survey response rates: a review. J Comput Commun.

[CR35] Strömbäck M, Wiklund M, Salander Renberg E, Malmgren-Olsson EB (2015). Complex symptomatology among young women who present with stress-related problems. Scand J Caring Sci.

[CR36] Willemsen G, Posthuma D, Boomsma DI (2005). Environmental factors determine where the Dutch live: results from the Netherlands twin register. Twin Res Hum Genet.

[CR37] Willemsen G, Vink JM, Abdellaoui A (2013). The adult Netherlands twin register: twenty-five years of survey and biological data collection. Twin Res Hum Genet.

[CR38] Yammarino FJ, Skinner SJ, Childers TL (1991). Understanding mail survey response behavior: a meta-analysis. Public Opin Q.

[CR39] Zasepa E, Wolanczyk T (2011). Assessment of problem behaviour in adults: evaluation of the psychometric properties of the polish adaptations of the adult self-report (ASR) and the adult behaviour checklist (ABCL). Int J Child Heal Hum Dev.

