# Supplementary material for: A Comparison of the ASEBA Adult Self Report (ASR) and the Brief Problem Monitor (BPM/18-59)
Source: Behav Genet. 2020 May 17;50(5):363–73. doi: 10.1007/s10519-020-10001-3 (PMC7441087; doi:10.1007/s10519-020-10001-3)
Supplement: Supplementary file 1 — Supplementary file1 (DOCX 66 kb) [file 10519_2020_10001_MOESM1_ESM.docx]

# Supplementary Material

**Table S1.** The correlations of the Adult Self Report (ASR) and Brief Problem Monitor (BPM) subscales with age. All confidence intervals of the comparable ASR and BPM subscales overlap, except for the Externalizing scales.

|  | ASR | BPM |
| --- | --- | --- |
| Total Problems | -0.164 *(-0.187, -0.142)* | -0.139 (-0.162, -0.117) |
| Internalizing Problems | -0.102 (-0.125, -0.079) | -0.085 (-0.108, -0.062) |
| Externalizing Problems | -0.166 (-0.189, -0.144) | -0.111 (-0.133, -0.088) |
| Attention Problems | -0.134 (-0.156, -0.111) | -0.149 (-0.171, -0.126) |

*Note*: 95% CIs are in parentheses.

**Table S2.** The means, standard deviations and variance of the Adult Self Report (ASR) and Brief Problem Monitor (BPM) subscales and subjective well-being, separately for the total sample and compared for men and women.

|  | |  | |  |  | | All | |  |  | Male |  | Female |  | Diff m/f |  |
| --- | --- | --- | --- | --- | --- | --- | --- | --- | --- | --- | --- | --- | --- | --- | --- | --- |
|  | *Weighted M* | | *Weighted SD* | | | *Weighted Var* | | *M* | *SD* | *Var* | *M* | *SD* | *M* | *SD* | *t* | *d* |
| ASR_TOT | | 0.27 | | 0.19 | 0.03 | | 31.47 | | 21.87 | 478.24 | 29.42 | 20.83 | 32.37 | 22.24 | -5.43* | 0.14 |
| ASR_INT | | 0.26 | | 0.25 | 0.06 | | 10.19 | | 9.07 | 82.23 | 8.19 | 8.02 | 11.06 | 9.36 | -13.26* | 0.32 |
| ASR_EXT | | 0.20 | | 0.17 | 0.03 | | 6.95 | | 5.85 | 34.17 | 7.23 | 5.99 | 6.83 | 5.78 | 2.68* | 0.07 |
| ASR_ATT | | 0.36 | | 0.29 | 0.08 | | 5.39 | | 4.28 | 18.29 | 5.14 | 4.20 | 5.51 | 4.31 | -3.40* | 0.09 |
|  | |  | |  |  | |  | |  |  |  |  |  |  |  |  |
| BPM_TOT | | 0.32 | | 0.28 | 0.08 | | 5.72 | | 4.98 | 24.78 | 5.09 | 4.62 | 5.99 | 5.10 | -7.30* | 0.18 |
| BPM_INT | | 0.30 | | 0.36 | 0.13 | | 1.77 | | 2.15 | 4.62 | 1.34 | 1.92 | 1.95 | 2.22 | -11.74* | 0.29 |
| BPM_EXT | | 0.28 | | 0.29 | 0.08 | | 1.66 | | 1.71 | 2.91 | 1.45 | 1.58 | 1.75 | 1.75 | -7.01* | 0.17 |
| BPM_ATT | | 0.38 | | 0.35 | 0.13 | | 2.29 | | 2.13 | 4.53 | 2.30 | 2.14 | 2.28 | 2.12 | 0.37 | 0.01 |
|  | |  | |  |  | |  | |  |  |  |  |  |  |  |  |
| SWB | |  | |  |  | | 27.20 | | 5.43 | 29.45 | 27.51 | 5.11 | 27.07 | 5.56 | 3.82* | 0.08 |

*Note*: N= 4040-4794. * p<.01. TOT= Total scale, INT= Internalizing scale, EXT= Externalizing scale, ATT= Attention scale, SWB= Subjective Well-Being, M= mean, SD= standard deviation, Var = variance.

**Table S3.** Proportion of participants in the clinical range and the agreement (Kappa estimate) between the scales per age and gender group.

| **Total** | ASR prop | BPM prop | Kappa | Z | n |
| --- | --- | --- | --- | --- | --- |
| All | 0.103 | 0.110 | 0.736 | 62.4 | 7169 |
| Man 18-35 | 0.100 | 0.113 | 0.749 | 28.1 | 1400 |
| Man >35 | 0.094 | 0.100 | 0.805 | 21.3 | 700 |
| Woman 18-35 | 0.108 | 0.111 | 0.731 | 41.1 | 3165 |
| Woman >35 | 0.103 | 0.112 | 0.709 | 28.8 | 1645 |
| **Internalizing** | ASR prop | BPM prop | Kappa | Z | n |
| All | 0.106 | 0.103 | 0.674 | 56.8 | 7092 |
| Man 18-35 | 0.097 | 0.094 | 0.701 | 26.1 | 1388 |
| Man >35 | 0.086 | 0.110 | 0.713 | 18.9 | 688 |
| Woman 18-35 | 0.117 | 0.102 | 0.681 | 38.3 | 3138 |
| Woman >35 | 0.108 | 0.112 | 0.646 | 26.0 | 1622 |
| **Externalizing** | ASR prop | BPM prop | Kappa | Z | n |
| All | 0.107 | 0.109 | 0.625 | 52.8 | 7129 |
| Man 18-35 | 0.116 | 0.118 | 0.620 | 23.2 | 1401 |
| Man >35 | 0.091 | 0.108 | 0.614 | 16.2 | 692 |
| Woman 18-35 | 0.100 | 0.098 | 0.648 | 36.3 | 3147 |
| Woman >35 | 0.122 | 0.124 | 0.609 | 24.6 | 1633 |
| **Attention** | ASR prop | BPM prop | Kappa | Z | n |
| All | 0.109 | 0.108 | 0.690 | 58.3 | 7136 |
| Man 18-35 | 0.099 | 0.107 | 0.683 | 25.6 | 1401 |
| Man >35 | 0.118 | 0.120 | 0.721 | 19.0 | 696 |
| Woman 18-35 | 0.117 | 0.098 | 0.678 | 38.2 | 3149 |
| Woman >35 | 0.097 | 0.120 | 0.699 | 28.4 | 1631 |

Note: ASR = Adult Self Report, BPM= Brief Problem Monitor, prop=proportion

**Table S4.** Model fitting results of the saturated univariate threshold twin models of the Adult Self Report (ASR) and Brief Problem Monitor (BPM) scores.

| **ASR** | Base | Comparison | ep | -2LL | df | AIC | p |
| --- | --- | --- | --- | --- | --- | --- | --- |
| Total | SAT |  | 11 | 10546.50 | 4954 | 638.50 |  |
|  | SAT | eqThresholdSex | 10 | 10556.36 | 4955 | 646.36 | 0.002 |
|  | SAT | eqThresholdTwin | 7 | 10560.74 | 4958 | 644.74 | 0.007 |
|  | SAT | eqThresholdZyg | 5 | 10567.26 | 4960 | 647.26 | 0.002 |
|  | SAT | ADE | 6 | 10575.80 | 4960 | 655.80 | 0.000 |
| Internalizing | SAT |  | 11 | 10058.47 | 4712 | 634.47 |  |
|  | SAT | eqThresholdSex | 10 | 10127.05 | 4713 | 701.05 | 0.000 |
|  | SAT | eqThresholdTwin | 7 | 10069.84 | 4716 | 637.84 | 0.023 |
|  | SAT | eqThresholdZyg | 5 | 10070.58 | 4718 | 634.58 | 0.060 |
|  | SAT | ADE | 6 | 10139.86 | 4718 | 703.86 | 0.000 |
| Externalizing | SAT |  | 11 | 10016.76 | 4683 | 650.76 |  |
|  | SAT | eqThresholdSex | 10 | 10026.23 | 4684 | 658.23 | 0.002 |
|  | SAT | eqThresholdTwin | 7 | 10018.76 | 4687 | 644.76 | 0.737 |
|  | SAT | eqThresholdZyg | 5 | 10028.87 | 4689 | 650.87 | 0.060 |
|  | SAT | ADE | 6 | 10039.77 | 4689 | 661.77 | 0.001 |
| Attention | SAT |  | 11 | 9744.61 | 4517 | 710.61 |  |
|  | SAT | eqThresholdSex | 10 | 9745.47 | 4518 | 709.47 | 0.352 |
|  | SAT | eqThresholdTwin | 7 | 9748.15 | 4521 | 706.15 | 0.472 |
|  | SAT | eqThresholdZyg | 5 | 9752.71 | 4523 | 706.71 | 0.231 |
|  | SAT | ADE | 6 | 9752.92 | 4523 | 706.92 | 0.216 |

| **BPM** | Base | Comparison | ep | -2LL | df | AIC | p |
| --- | --- | --- | --- | --- | --- | --- | --- |
| Total | SAT |  | 11 | 9529.89 | 4412 | 705.89 |  |
|  | SAT | eqThresholdSex | 10 | 9547.52 | 4413 | 721.52 | 0.000 |
|  | SAT | eqThresholdTwin | 7 | 9539.14 | 4416 | 707.14 | 0.055 |
|  | SAT | eqThresholdZyg | 5 | 9546.23 | 4418 | 710.23 | 0.012 |
|  | SAT | ADE | 6 | 9563.83 | 4418 | 727.83 | 0.000 |
| Internalizing | SAT |  | 11 | 10359.47 | 4906 | 547.47 |  |
|  | SAT | eqThresholdSex | 10 | 10439.14 | 4907 | 625.14 | 0.000 |
|  | SAT | eqThresholdTwin | 7 | 10362.83 | 4910 | 542.83 | 0.500 |
|  | SAT | eqThresholdZyg | 5 | 10362.89 | 4912 | 538.89 | 0.750 |
|  | SAT | ADE | 6 | 10456.09 | 4912 | 632.09 | 0.000 |
| Externalizing | SAT |  | 11 | 7268.50 | 3372 | 524.50 |  |
|  | SAT | eqThresholdSex | 10 | 7279.11 | 3373 | 533.11 | 0.001 |
|  | SAT | eqThresholdTwin | 7 | 7276.03 | 3376 | 524.03 | 0.110 |
|  | SAT | eqThresholdZyg | 5 | 7289.47 | 3378 | 533.47 | 0.001 |
|  | SAT | ADE | 6 | 7302.86 | 3378 | 546.86 | 0.000 |
| Attention | SAT |  | 11 | 8036.80 | 3715 | 606.80 |  |
|  | SAT | eqThresholdSex | 10 | 8037.52 | 3716 | 605.52 | 0.395 |
|  | SAT | eqThresholdTwin | 7 | 8040.26 | 3719 | 602.26 | 0.483 |
|  | SAT | eqThresholdZyg | 5 | 8050.20 | 3721 | 608.20 | 0.037 |
|  | SAT | ADE | 6 | 8051.82 | 3721 | 609.82 | 0.020 |

*Note*: SAT= Saturated model, ep= estimated parameters, eqThresholdSex = equal thresholds in males and females, eqThresholdTwin = equal thresholds in twin 1 and twin 2, eqThresholdZyg= equal thresholds in monozygotic and dizygotic twins.

**Table S5.** Model fitting results of the univariate threshold twin models of the Adult Self Report (ASR) and Brief Problem Monitor (BPM) scores.

| **ASR** | Base | Comp | ep | -2LL | df | AIC | p |
| --- | --- | --- | --- | --- | --- | --- | --- |
| Total | ADE |  | 6 | 10575.80 | 4960 | 655.80 |  |
|  | ADE | **AE** | 5 | 10576.37 | 4961 | 654.37 | 0.451 |
|  | ADE | E | 4 | 10894.66 | 4962 | 970.66 | 0.000 |
| Internalizing | ADE |  | 6 | 10139.86 | 4718 | 703.86 |  |
|  | ADE | **AE** | 5 | 10139.86 | 4719 | 701.86 | 0.999 |
|  | ADE | E | 4 | 10356.65 | 4720 | 916.65 | 0.000 |
| Externalizing | ADE |  | 6 | 10039.77 | 4689 | 661.77 |  |
|  | ADE | **AE** | 5 | 10039.99 | 4690 | 659.99 | 0.644 |
|  | ADE | E | 4 | 10212.96 | 4691 | 830.96 | 0.000 |
| Attention | ADE |  | 6 | 9752.92 | 4523 | 706.92 |  |
|  | ADE | **AE** | 5 | 9754.91 | 4524 | 706.91 | 0.159 |
|  | ADE | E | 4 | 9928.37 | 4525 | 878.37 | 0.000 |

| **BPM** | Base | Comp | ep | -2LL | df | AIC | p |
| --- | --- | --- | --- | --- | --- | --- | --- |
| Total | ADE |  | 6 | 9563.83 | 4418 | 727.83 |  |
|  | ADE | **AE** | 5 | 9564.77 | 4419 | 726.77 | 0.333 |
|  | ADE | E | 4 | 9707.67 | 4420 | 867.67 | 0.000 |
| Internalizing | ADE |  | 6 | 10456.09 | 4912 | 632.09 |  |
|  | ADE | **AE** | 5 | 10459.64 | 4913 | 633.64 | 0.060 |
|  | ADE | E | 4 | 10677.57 | 4914 | 849.57 | 0.000 |
| Externalizing | ADE |  | 6 | 7302.86 | 3378 | 546.86 |  |
|  | ADE | **AE** | 5 | 7304.82 | 3379 | 546.82 | 0.161 |
|  | ADE | E | 4 | 7352.84 | 3380 | 592.84 | 0.000 |
| Attention | ADE |  | 6 | 8051.82 | 3721 | 609.82 |  |
|  | ADE | **AE** | 5 | 8053.29 | 3722 | 609.29 | 0.226 |
|  | ADE | E | 4 | 8136.05 | 3723 | 690.05 | 0.000 |

*Note*: A = additive genetic, D = non-additive genetic, E = non-shared environmental influences including measurement error, Comp= Comparison, ep= estimated parameters.

**Table S6.** Standardized estimates (95 % CI) for the additive genetic, non-additive genetic and non-shared environmental influences on the Adult Self Report (ASR) and Brief Problem Monitor (BPM), based on the full ADE model and the best fitting model (AE model).

|  | | ASR | |  | | | BPM | |  | |
| --- | --- | --- | --- | --- | --- | --- | --- | --- | --- | --- |
|  | A | | D | | E | A | | D | | E |
| TOT | .421 (.112, .579) | | .121 (.000, .441) | | .459 (.410, .511) | .246 (0, .469) | | .182 (0, .487) | | .573 (.510, .638) |
| INT | .472 (.152, .525) | | .000 (.000, .000) | | .528 (.473, .581) | .157 (0, .476) | | .321 (0, .528) | | .522 (.469, .579) |
| EXT | .359 (.020, .491) | | .082 (.006, .437) | | .559 (.500, 621) | .001 (0, .372) | | .338 (0, .422) | | .662 (.578, .753) |
| ATT | .211 (.000, .481) | | .246 (.213, .508) | | .544 (.485, .606) | .131 (0, 430) | | .260 (0, .465) | | .609 (.535, .689) |

|  | ASR | | BPM | |
| --- | --- | --- | --- | --- |
|  | A | E | A | E |
| TOT | .535 (.485, .582) | .465(.418, .515) | .416 (.354, .476) | .584 (.524, .647) |
| INT | .472 (.416, .525) | .528 (.475, .584) | .461 (.406, .513) | .539 (.487-.594) |
| EXT | .436 (.377, .493) | .564 (.507, 623) | .314 (.228, .396) | .686 (.604, 772) |
| ATT | .441 (.000, .481) | .559 (.485, .606) | .373 (.297, .445) | .627 (.555, .703) |

*Note*: TOT= Total scale, INT= Internalizing scale, EXT= Externalizing scale, ATT= Attention scale, A = additive genetic, D = non-additive genetic, E = non-shared environmental influences including measurement error. 95% CIs are in parentheses.

**Table S7.** The phenotypic, genetic (rA) and environmental (rE) correlations between the Adult Self Report (ASR), Brief Problem Monitor (BPM) and subjective well-being scores (SWB), based on the best fitting model (AE models).

|  | **ASR/SWB** | | | | **BPM/SWB** | | |  |
| --- | --- | --- | --- | --- | --- | --- | --- | --- |
|  | phenotypic | rA | rE | phenotypic | | rA | rE | |
| TOT | -.492  (-.509, -.474) | -.492  (-.572, -.409) | -.372  (-.433, -.308) | -.478  (-.496, -.460) | | -.481  (-.576, -.465) | -.375  (-.438, -.311) | |
| INT | -.561  (-.577, -.545) | -.581  (-.664, -.494) | -.394  (-.453, -.331) | -.536  (-.552, -.519) | | -.589  (-.631, -.580) | -.411  (-.460, -.352) | |
| EXT | -.311  (-.332, -.290) | -.257  (-.360, -.213) | -.247  (-.281, -.204) | -.303  (-.323, -.281) | | -.266  (-.331, -.206) | -.224  (-.281, -.180) | |
| ATT | -.347  (-.368, -.327) | -.339  (-.428, -.292) | -.273  (-.314, -.227) | -.334  (-.355, -.314) | | -.382  (-.418, -.256) | -.237  (-.310, -.161) | |

*Note*: TOT= Total scale, INT= Internalizing scale, EXT= Externalizing scale, ATT= Attention scale, rA = genetic correlation, rE = environmental correlations. 95% CIs are in parentheses.

**Table S8.** The regression estimates for the Adult Self Report (ASR)/Brief Problem Monitor (BPM) scores on subjective well-being.

| Scale | *Estimate* (95% CI) | *SE* | *Z* |
| --- | --- | --- | --- |
| ASR Total | -0.499 (-0.525, -0.473) | 0.013 | -37.49* |
| BPM Total | -0.485 (-0.512, -0.459) | 0.013 | -38.49* |
| ASR Internalizing | -0.569 (-0.593, -0.544) | 0.013 | -44.67* |
| BPM Internalizing | -0.545 (-0.570, -0.520) | 0.013 | -43.35* |
| ASR Externalizing | -0.316 (-0.343, -0.288) | 0.014 | -22.57* |
| BPM Externalizing | -0.307 (-0.335, -0.280) | 0.014 | -22.74* |
| ASR Attention | -0.353 (-0.379, -0.327) | 0.013 | -26.66* |
| BPM Attention | -0.339 (-0.365, -0.313) | 0.013 | -25.49* |

Note: * p<.001. 95% CIs are in parentheses.

**Table S9.** Model fitting results of the saturated bivariate threshold twin models of the Adult Self-Report (ASR) and Brief Problem Monitor (BPM) scores with subjective well-being (SWB).

| **ASR** | Base | Comparison | ep | minus2LL | df | AIC | p |
| --- | --- | --- | --- | --- | --- | --- | --- |
| SWB/Total | SAT |  | 30 | 23277.93 | 11209 | 859.93 |  |
|  | SAT | eqThresholdSex | 28 | 23292.59 | 11211 | 870.59 | 0.001 |
|  | SAT | eqThresTwin | 22 | 23334.36 | 11217 | 900.36 | 0.000 |
|  | SAT | eqThresZyg | 18 | 23342.82 | 11221 | 900.82 | 0.000 |
|  | SAT | ADE | 13 | 23358.66 | 11228 | 902.66 | 0.000 |
| SWB/Internalizing | SAT |  | 30 | 22681.49 | 10967 | 747.49 |  |
|  | SAT | eqThresholdSex | 28 | 22754.73 | 10969 | 816.73 | 0.000 |
|  | SAT | eqThresTwin | 22 | 22725.99 | 10975 | 775.99 | 0.000 |
|  | SAT | eqThresZyg | 18 | 22727.39 | 10979 | 769.39 | 0.000 |
|  | SAT | ADE | 13 | 22805.47 | 10986 | 833.47 | 0.000 |
| SWB/Externalizing | SAT |  | 30 | 23125.91 | 10940 | 1245.91 |  |
|  | SAT | eqThresholdSex | 28 | 23148.89 | 10942 | 1264.89 | 0.000 |
|  | SAT | eqThresTwin | 22 | 23169.41 | 10948 | 1273.41 | 0.000 |
|  | SAT | eqThresZyg | 18 | 23182.04 | 10952 | 1278.04 | 0.000 |
|  | SAT | ADE | 13 | 23206.99 | 10959 | 1288.99 | 0.000 |
| SWB/Attention | SAT |  | 30 | 22794.88 | 10774 | 1246.88 |  |
|  | SAT | eqThresholdSex | 28 | 22803.68 | 10776 | 1251.68 | 0.012 |
|  | SAT | eqThresTwin | 22 | 22835.44 | 10782 | 1271.44 | 0.000 |
|  | SAT | eqThresZyg | 18 | 22840.69 | 10786 | 1268.69 | 0.000 |
|  | SAT | ADE | 13 | 22851.01 | 10793 | 1265.01 | 0.000 |

| **BPM** | Base | Comparison | ep | minus2LL | df | AIC | p |
| --- | --- | --- | --- | --- | --- | --- | --- |
| SWB/Total | SAT |  | 30 | 22332.44 | 10670 | 992.44 |  |
|  | SAT | eqThresholdSex | 28 | 22353.03 | 10672 | 1009.03 | 0.000 |
|  | SAT | eqThresTwin | 22 | 22383.55 | 10678 | 1027.55 | 0.000 |
|  | SAT | eqThresZyg | 18 | 22390.70 | 10682 | 1026.70 | 0.000 |
|  | SAT | ADE | 13 | 22417.19 | 10689 | 1039.19 | 0.000 |
| SWB/Internalizing | SAT |  | 30 | 22896.69 | 11161 | 574.69 |  |
|  | SAT | eqThresholdSex | 28 | 22977.06 | 11163 | 651.06 | 0.000 |
|  | SAT | eqThresTwin | 22 | 22939.19 | 11169 | 601.19 | 0.000 |
|  | SAT | eqThresZyg | 18 | 22939.91 | 11173 | 593.91 | 0.000 |
|  | SAT | ADE | 13 | 23048.1 | 11180 | 688.1032 | 0.000 |
| SWB/Externalizing | SAT |  | 30 | 20463.35 | 9634 | 1195.35 |  |
|  | SAT | eqThresholdSex | 28 | 20480.83 | 9636 | 1208.83 | 0.000 |
|  | SAT | eqThresTwin | 22 | 20510.98 | 9642 | 1226.98 | 0.000 |
|  | SAT | eqThresZyg | 18 | 20524.37 | 9646 | 1232.37 | 0.000 |
|  | SAT | ADE | 13 | 20545.12 | 9653 | 1239.115 | 0.000 |
| SWB/Attention | SAT |  | 30 | 21153.66 | 9976 | 1201.66 |  |
|  | SAT | eqThresholdSex | 28 | 21158.28 | 9978 | 1202.28 | 0.100 |
|  | SAT | eqThresTwin | 22 | 21190.07 | 9984 | 1222.07 | 0.000 |
|  | SAT | eqThresZyg | 18 | 21198.80 | 9988 | 1222.80 | 0.000 |
|  | SAT | ADE | 13 | 21254.80 | 9995 | 1222.80 | 0.000 |

*Note:* Base= baseline model, SAT= Saturated model, ep= estimated parameters, eqThresholdSex = equal thresholds in males and females, eqThresholdTwin = equal thresholds in twin 1 and twin 2, eqThresholdZyg= equal thresholds in monozygotic and dizygotic twins.

**Table S10.** Model fitting results of the bivariate threshold twin models of the Adult Self-Report (ASR) and Brief Problem Monitor (BPM) scores with subjective well-being (SWB).

| **ASR** | Base | Comp | ep | -2LL | df | AIC | p |
| --- | --- | --- | --- | --- | --- | --- | --- |
| SWB/Total | ADE |  | 13 | 23485.38 | 11290 | 905.38 |  |
|  | ADE | **AE** | 10 | 23486.45 | 11293 | 900.44 | 0.786 |
|  | ADE | E | 7 | 23962.73 | 11296 | 1370.73 | 0.000 |
| SWB/Internalizing | ADE |  | 13 | 22930.85 | 11048 | 834.85 |  |
|  | ADE | **AE** | 10 | 22930.85 | 11051 | 828.85 | 1.000 |
|  | ADE | E | 7 | 23305.33 | 11054 | 1197.33 | 0.000 |
| SWB/Externalizing | ADE |  | 13 | 23333.60 | 11019 | 1295.60 |  |
|  | ADE | **AE** | 10 | 23333.99 | 11022 | 1289.98 | 0.943 |
|  | ADE | E | 7 | 23692.66 | 11025 | 1642.66 | 0.000 |
| SWB/Attention | ADE |  | 13 | 22972.17 | 10853 | 1266.17 |  |
|  | ADE | **AE** | 10 | 22974.11 | 10856 | 1262.11 | 0.589 |
|  | ADE | E | 7 | 23326.33 | 10859 | 1608.32 | 0.000 |

| **BPM** | Base | Comp | ep | -2LL | df | AIC | p |
| --- | --- | --- | --- | --- | --- | --- | --- |
| SWB/Total | ADE |  | 13 | 22541.83 | 10748 | 1045.82 |  |
|  | ADE | **AE** | 10 | 22418.47 | 10751 | 1040.99 | 0.761 |
|  | ADE | E | 7 | 22743.89 | 10754 | 1359.85 | 0.000 |
| SWB/Internalizing | ADE |  | 13 | 23171.89 | 11242 | 687.89 |  |
|  | ADE | **AE** | 10 | 23176.10 | 11245 | 686.10 | 0.239 |
|  | ADE | E | 7 | 23543.02 | 11248 | 1047.02 | 0.000 |
| SWB/Externalizing | ADE |  | 13 | 20660.26 | 9709 | 1244.26 |  |
|  | ADE | **AE** | 10 | 20663.08 | 9711 | 1241.08 | 0.420 |
|  | ADE | E | 7 | 20899.58 | 9714 | 1471.58 | 0.000 |
| SWB/Attention | ADE |  | 13 | 21331.36 | 10051 | 1229.36 |  |
|  | ADE | **AE** | 10 | 21333.62 | 10054 | 1225.62 | 0.521 |
|  | ADE | E | 7 | 21604.55 | 10057 | 1490.55 | 0.000 |

*Note*: Base = baseline model. A = additive genetic, D = non-additive genetic, E = non-shared environmental influences including measurement error, Comp= Comparison, ep= estimated parameters.

**Table S11.** The bivariate estimates (95% CI) for subjective well-being (SWB) and the Adult Self-Report (ASR) and Brief Problem Monitor (BPM) scores.

|  | ASR | | BPM | |
| --- | --- | --- | --- | --- |
|  | A | E | A | E |
| WB/TOT | 0.520 (.431, .606) | 0.480 (.380, .570) | 0.461 (.435, .546) | 0.539 (.454, .577) |
| WB/INT | 0.517 (433, .598) | 0.483 (.403, .567) | 0.503 (.428, .534) | 0.497 (.419, .572) |
| WB/EXT | 0.414 (.341, .570) | 0.586 (.430, .660) | 0.387 (.356, 480) | 0.613 (.519, .664) |
| WB/ATT | 0.456 (.317, .523) | 0.544 (.462, .615) | 0.491 (.334, .646) | 0.509 (.354, .666) |

*Note*: TOT= Total scale, INT= Internalizing scale, EXT= Externalizing scale, ATT= Attention scale, A = additive genetic, E = non-shared environmental influences including measurement error. 95% CIs are in parentheses.
